# Can We Predict the Isosymmetric Phase Transition? Application of DFT Calculations to Study the Pressure Induced Transformation of Chlorothiazide

**DOI:** 10.3390/ijms221810100

**Published:** 2021-09-18

**Authors:** Łukasz Szeleszczuk, Anna Helena Mazurek, Katarzyna Milcarz, Ewa Napiórkowska, Dariusz Maciej Pisklak

**Affiliations:** 1 Department of Physical Chemistry, Chair and Department of Physical Pharmacy and Bioanalysis, Faculty of Pharmacy, Medical University of Warsaw, Banacha 1 Street, 02-093 Warsaw, Poland; kmilcarz@wum.edu.pl (K.M.); enapiorkowska@wum.edu.pl (E.N.); dpisklak@wum.edu.pl (D.M.P.); 2 Department of Physical Chemistry, Chair and Department of Physical Pharmacy and Bioanalysis, Faculty of Pharmacy, Doctoral School, Medical University of Warsaw, Banacha 1 Street, 02-093 Warsaw, Poland; anna.mazurek@wum.edu.pl

**Keywords:** DFT, CASTEP, aiMD, ab initio molecular dynamics, phase transition, polymorphism

## Abstract

Isosymmetric structural phase transition (IPT, type 0), in which there are no changes in the occupation of Wyckoff positions, the number of atoms in the unit cell, and the space group symmetry, is relatively uncommon. Chlorothiazide, a diuretic agent with a secondary function as an antihypertensive, has been proven to undergo pressure-induced IPT of Form I to Form II at 4.2 GPa. For that reason, it has been chosen as a model compound in this study to determine if IPT can be predicted in silico using periodic DFT calculations. The transformation of Form II into Form I, occurring under decompression, was observed in geometry optimization calculations. However, the reverse transition was not detected, although the calculated differences in the DFT energies and thermodynamic parameters indicated that Form II should be more stable at increased pressure. Finally, the IPT was successfully simulated using ab initio molecular dynamics calculations.

## 1. Introduction

Polymorphism, commonly defined as the ability of a substance to exist as two or more crystalline phases that have different arrangements or conformations of the molecules in the crystal lattice [1] is a phenomenon with particular importance in the pharmaceutical sciences and industry. The differences between polymorphs at the molecular level can manifest themselves in different properties, important in this field, such as hygroscopicity, solubility, thermal stability, rate of dissolution, hardness, chemical reactivity, and many others [2,3]. For the correct design of a pharmaceutical compound, it is, therefore, crucial to control its solid-state form to guarantee its properties.

The application of high pressure has been shown as a route to access new phases of solid-state materials—possibly the most famous example being the transformation of graphite into diamond [4]. Under increased pressure, the geometry of both inter and intramolecular bonds can often be altered, new hydrogen bonds can be formed, and existing ones broken or symmetrized. In most cases, pressure-induced phase transition can occur in a single step between higher- and lower-symmetry space groups (type I), through a low-symmetry transition state between relatively higher- symmetry initial and final structures (type II), or via the transformation in which the mechanism is more complex (type III). Isosymmetric structural phase transition (IPT, type 0), in which there are no changes in the occupation of Wyckoff positions, the number of atoms in the unit cell, and the space group symmetry are relatively uncommon [5]. However, there are some well-known and recently discovered examples of such transitions: sodium oxalate [6], 1,3-cyclohexandione [7] L-serine [8], sulfamic acid [9], biurea [10], and α-glycylglycine [11].

The density functional theory (DFT) methods are commonly used to model the structure and properties of organic molecules. However, the uniqueness of each polymorphic form property arises mostly from short- and long-distance intermolecular interactions. Therefore, DFT-based methods in which a single molecule in vacuum or in solution is being modeled were found to be inappropriate and inaccurate to study the polymorphism-related phenomena. While those “single molecules” methods are generally successfully applied in other aspects of pharmaceutical sciences, i.e., to study drug–biomolecule interactions or to predict the formation of complexes, in order to study the solid-state pharmaceutics, other types of calculations, sometimes called “periodic DFT calculations”, should be used [12,13,14]. In this case, the adjective “periodic” is an abbreviation of “performed under periodic boundary conditions” which is a crucial requirement for accurate modeling of crystals. Further, in such calculations, the pseudopotentials are frequently used to represent an effective interaction that approximates the potential experienced by the valence electrons. Additionally, the plane-wave basis sets are employed instead of the localized ones [15,16].

Noncovalent forces, such as hydrogen bonding and van der Waals interactions, are crucial for the formation, stability, and function of molecules and materials [17,18]. There exists a variety of hybrid semiempirical solutions that introduce dispersion corrections in the DFT formalism [19]. These semiempirical approaches provide the best compromise between the cost of the first principals evaluation of the dispersion terms and the need to improve non-bonding interactions in the standard DFT description. The Tkatchenko–Scheffler (TS) correction [20], used in this study, exploits the relationship between polarizability and volume, and thus accounts to some degree for the relative variation in dispersion coefficients of differently bonded atoms. This is achieved by weighting values taken from the high-quality first-principals database with atomic volumes derived from Hirshfeld partitioning of the self-consistent electronic density. It should be noted, however, that the TS scheme is an atom-pairwise dispersion model. As dispersion interactions are not strictly pairwise additive, many-body dispersion interactions can become important for some systems, especially when large and flexible molecules are involved [21]. Such interactions can be captured by the many-body dispersion (MBD) model [22]. However, the object of this study, chlorothiazide, is a rather small and rigid molecule, therefore, only small differences between the TS and MBD dispersion models would have been expected. The increasing number of studies presenting results of periodic DFT calculations on pharmaceutical solids confirms that those kinds of computations can be successfully used to answer the fundamental questions as well as to provide specific solutions for experimental challenges. Many successful applications of such calculations have been recently reviewed by us [23]. However, among those works, there was no application of periodic DFT calculations to study the phenomenon of isosymmetric phase transition in the manner presented in this study.

To be precise, it must be stated that some of the crystals that have been proven experimentally to undergo IPT were also modeled using the DFT methods. However, in those cases, the computational part was limited to the geometry optimization at the pressure at which the particular structure was obtained, followed sometimes by the thermodynamic parameters calculations [10,11].

However, presently, we have decided to expand the spectrum of the applied computational methods by using the various DFT functionals, empirical dispersion corrections, phonon density of states calculations, and computationally demanding ab initio molecular dynamics calculations (aiMD). This effort has been made to answer the question if such IPT can be predicted and, if yes, how it should be carried out. To the best of our knowledge, there are no published works presenting an application of such combined calculations to study the phenomenon of IPT.

**Figure 1:**
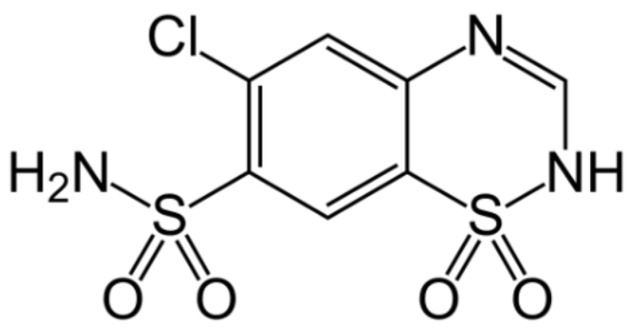
Chemical structure of chlorothiazide (CT).

**Figure 2:**
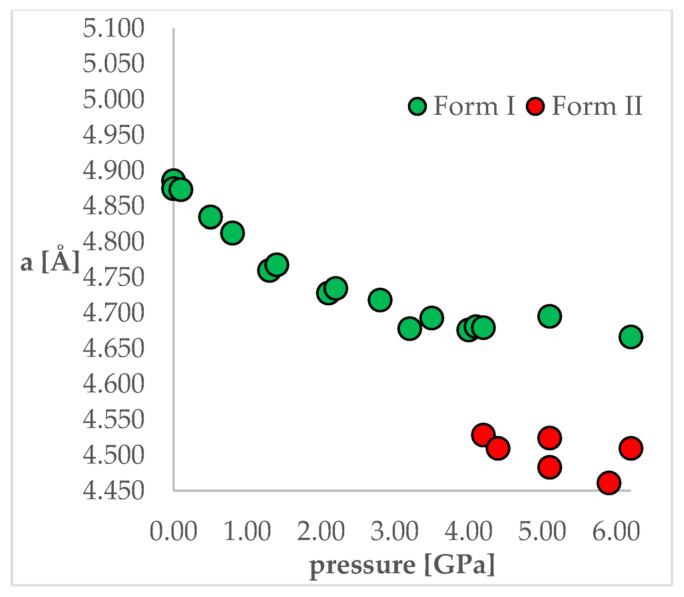
The change of unit cell edge “a” length with respect to pressure. Green circles—Form I, Red circles—Form II.

To achieve this aim, chlorothiazide (6-chloro-4H-1,2,4-benzothiadiazine-7-sulfonamide 1,1-dioxide, CT, Figure 1) has been chosen as a model compound. This active pharmaceutical ingredient (API) is used as a diuretic agent with a secondary function as an antihypertensive [24]. It exists in various complex solid-state forms, including solvates and co-crystals. However, at ambient conditions, only one polymorphic form of CT has so far been reported (Form I). More importantly, this API has been chosen as its crystal structure and pressure-induced IPT have been studied experimentally by Oswald et al. [25]. In that work, the authors obtained a series of crystal structures of CT at various pressure conditions and confirmed that the IPT occurs at 4.2 GPa resulting in Form II, which was found to be more stable at pressures higher than 4.2 GPa. For CT, the most noticeable differences between Forms I and II studied at the same conditions can be observed for “a” length of a unit cell, which is clearly visible in Figure 2. Unit cell dimensions of all studied structures can be found in Table 1.

As the aim of the study was to also check the accuracy of the applied computational methods, it was important that this coherent group of crystal structures was obtained in one study as it allowed the elimination of the possible differences in the unit cell dimensions caused by the application of different diffractometers or diamond anvil cells used for high-pressure experiments. This would be an issue if the structures originated from different works. From Figure 2 and Table 1, it can be observed that some changes of the unit cell dimensions are non-monotonic upon compression. This was probably caused by inaccuracies in their determinations as, at some of the studied pressures, the authors performed solely powder X-ray diffraction (PXRD) measurements followed by pattern refinement.

Having the appropriate amount of structural data, clearly showing the IPT of CT, there was nothing left but to check how the periodic DFT calculations performed in such cases. Our motivation was that if we succeed with this model compound, this method can be further validated on other solid organics undergoing IPT and could finally be used as a screening method in order to predict if the pressure-driven IPT would occur for a particular compound, assuming that only the low-pressure structure is known. Such a method would surely help to design the demanding high-pressure experiments.

## 2. Results and Discussion

### 2.1. Choice of the DFT Functionals

The first set of calculations (Table S1) was performed to find out how the choice of the DFT functional would affect the accuracy of geometry optimization and to choose the most accurate one for the subsequent calculations. This was carried out by the optimization of the experimental crystal structure obtained at normal conditions (refcode QQQAUG09). This step is usually omitted in the studies presenting the results of DFT calculations on crystals. This is because, similarly to B3LYP for in vacuo calculations, the GGA PBE functional with TS dispersion correction has not been proved many times to be the most accurate in the case of solid-phase modeling. Unsurprisingly, also in this study, the most accurate results have been obtained by the GGA PBE TS approach. Using this functional, the differences between the experimental and calculated unit cell dimensions were lower than 0.05 Å for lengths and 0.5 ° for angles, while for the other functionals those differences were in some cases found to be larger than 1 Å and 10.

However, in addition to the GGA PBE TS, we have also decided to choose the PBESOL. Although, without any dispersion correction, this functional was specially designed and validated for the densely packed solids, and was in some cases shown to be more accurate than GGA PBE TS, especially for the calculations performed under increased pressure [26].

### 2.2. Geometry Optimization of Forms I and II under External Pressure—Unit Cell Dimensions Analysis

**Figure 3:**
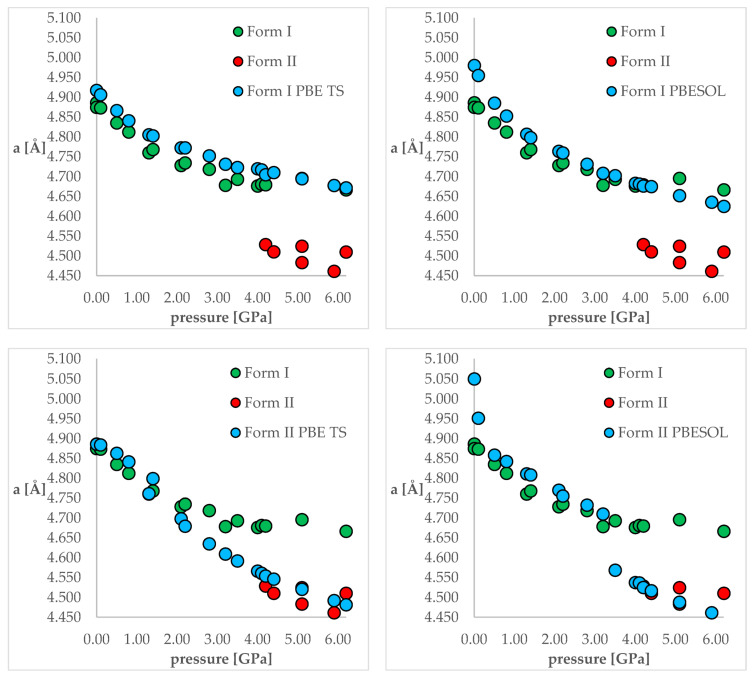
The change of unit cell edge “a” length with respect to pressure. Green circles—experimental Form I; red circles—experimental Form II; blue circles—calculated ones. **Top left**—using PBE TS and starting from Form I; **top right**—using PBESOL and starting from Form I; **bottom left**—using PBE TS and starting from Form II; **top right**—using PBESOL and starting from Form II.

The next stage of this work was the optimization of both polymorphic forms (I and II) under external pressure to find out if the IPT can be observed. More specifically, we have chosen two crystal structures of CT—the structure of Form I obtained at normal conditions (QQQAUG09) and Form II obtained at 5.9 GPa (QQQAUG17). These structures were obtained at the most distinct pressure conditions. Both structures were optimized at 19 different values of external pressure, exactly those that had been applied experimentally, listed in Table 1. Due to the large number of calculations (two crystal structures, two DFT functionals, 18 values of pressure = 72 optimizations), the obtained unit cell dimensions are listed in Tables S2 and S3 for clarity reasons. Additionally, the visual representation of some of the results are presented in Figure 3. Below, firstly, the changes in the unit cell dimensions upon compression (when geometry optimization starting from Form I) or decompression (when geometry optimization starting from Form II) will be discussed. Then, the RMSD of the calculated structures will be presented and analyzed. Finally, the differences in the energies calculated at the same pressure and using the same DFT functionals but different structures will be discussed.

Looking at Figure 3, it is clearly visible that both choice of the DFT functional and of the initial structure (polymorphic form) subjected to geometry optimization had an influence on the obtained results. For the calculations of Form I (top left and top right), the results obtained using PBE TS were more accurate than those obtained using PBESOL, especially for lower pressure values (0 and 0.1 GPa). Application of neither of those functionals enables prediction of the IPT during the compression process because even at higher pressures Form I was still preserved after optimization.

More interesting were the results obtained through modeling of the decompression, which is when Form II had been chosen as the starting one (bottom left and bottom right). For the PBE TS calculations, Form II was preserved, though it is impossible to assess the accuracy of those calculations as Form II was not obtained at pressures lower than 4.2 GPa. However, the changes in the unit cell dimensions were continuous, which suggested that there should be no IPT. However, when the calculations were performed using PBESOL, Form II was preserved only for pressures higher than 3.5 GPa. At this pressure, a jump discontinuity can be observed, which would suggest that at this point one can expect IPT. As an experimentally determined pressure at which IPT occurs was found to be 4.2 GPa, there was a 0.7 GPa difference between the experimental and theoretical values. However, it should be noticed that the experiments were carried out at 293 K while the geometry optimization calculations were performed at 0 K. Therefore, neglecting the thermal motions could be the main reason for this inaccuracy. Nevertheless, DFT calculations of the decompression of Form II using PBESOL enable the prediction that IPT would occur. Unfortunately, in most cases, the low-pressure crystal structure of a compound is known while the question remains if compression would result in the IPT. In the case of CT, this question could not have been answered using solely energy minimization. However, this did not discourage us to look for another solution which will be described in one of the next paragraphs (2.5).

### 2.3. Geometry Optimization of Forms I and II under External Pressure—RMSD Analysis

So far, only the differences between the experimental and calculated unit cell dimensions were discussed. However, to confirm the results discussed above, the analysis of the conformational changes should also be performed. Though CT is an API with rather limited conformational space, there are of course some conformational differences between the molecules found in Form I and Form II. Instead of presenting the comparison of the lengths, angles, and dihedrals, we have decided to calculate the root mean square deviation (RMSD) values between the calculated and experimental crystal structures. This analysis was, however, hampered by the lack of some experimental data. As is presented in Table 1, only part of the structural parameters originated from the SCXRD measurements while the rest from PXRD. The crystal structures have been fully solved, including the determination of the positions of the atoms, only for structures studied by SCXRD. Besides, there was no pressure at which the SCXRD has been done for both Form I and Form II. Therefore, the RMSD analysis was limited to nine examples (Table 2 A–I).

The coloring scheme for the values in Table 2A–I was applied to facilitate their categorization into five groups (A and B; C and D; E and F; G, H, and I) in order of increasing pressure. In the first group (A and B), the most accurate results were obtained when PBE TS functional was used for optimization, which is also consistent with results presented in Table S1. Besides, the choice of the initial structure had no influence on the obtained results. In other words, no matter whether Form I or Form II was used as the initial structure, after geometry optimization, an accurate structure of Form I was obtained. However, in the case of PBESOL calculations, the choice of the initial structure had an influence on the obtained results. Though PBESOL, as described previously, was found to be less accurate than PBE TS for the calculations at 0 GPa, more accurate results were obtained for Form I than II (RMSD 0.1249 vs. 0.1724).

Quite opposite results could be observed in the second group (C and D). This time, the accuracy of the results obtained using PBESOL and PBE TS was almost the same, with one important observation. For the PBESOL, the choice of the initial form had no influence on the results, while for the PBE TS the accurate results were obtained only if the proper form (I) had been chosen as the initial structure.

For the third group (E and F), the accuracy of PBE TS and PBESOL was almost the same. However, this time, the choice of the correct initial form (I) was crucial to obtain accurate results for both functionals. This is also elegantly reflected in Figure 3, which shows that the length of “a” edge obtained after optimization at 3.5 and 4.0 GPa depends significantly on the initial structure but not on the DFT functional used. Those results correspond nicely with experimental ones, as for those pressure values Forms I and II were found to coexist. This has been proven by the PXRD analysis, as in the diffractograms recorded at those conditions peaks from both Form I and Form II could have been observed. This may suggest that the Gibbs free energies of those two polymorphs are very similar, and thus if the phase transition had occurred during optimization, it would have been associated with a negligible change of the free energy.

The last analyzed group (G, H, and I) is also very consistent in terms of the received RMSD values. For this group, unlike for the others, Form II was the experimentally obtained one, which resulted in small RMSD values for comparisons between the experimental and modeled structures when Form II was used as the initial structure for calculations. As previously stated, based on the results presented in Tables S2 and S3 and Figure 3, the application of neither PBE TS nor PBESOL enabled the prediction of the ISP to obtain Form II while optimizing Form I at the increased pressure. This is also reflected in the large RMSD values between the experimental structure (Form II) and calculated ones, using Form I as the initial for both PBESOL and PBE TS. For this group, the differences between the results obtained using PBE TS and PBESOL were found to be negligible when either Form I or Form II was used as the initial.

### 2.4. Geometry Optimization of Forms I and II under External Pressure—Energy and Thermodynamic Parameters Differences Analysis

**Figure 4:**
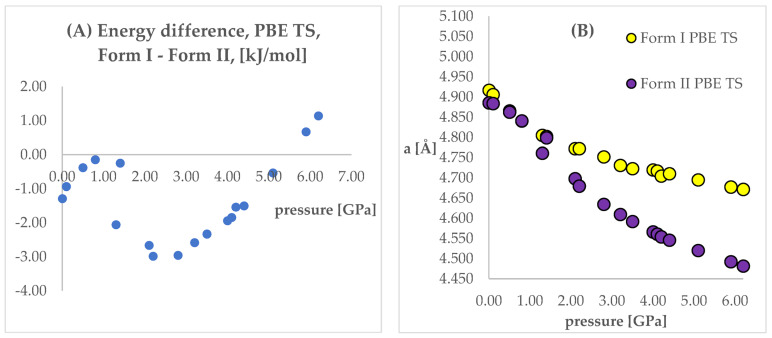
(**A**): Differences between the energies (Form I—Form II) of the structures modeled using PBE TS functional, with respect to pressure. (**B**): The change of unit cell edge “a” length obtained from calculations using PBE TS functional, with respect to pressure. Yellow circles—using Form I as initial; violet circles—using Form II as initial.

**Figure 5:**
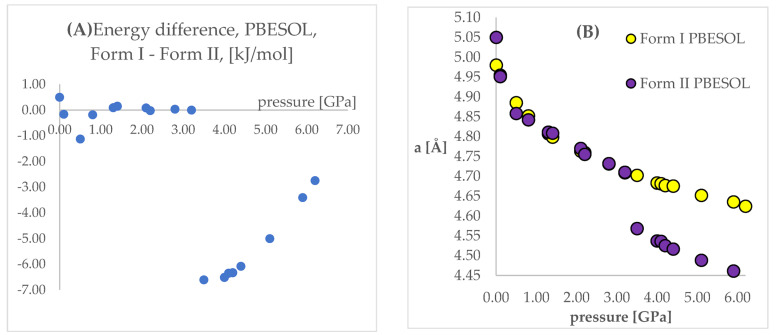
(**A**): Differences between the energies (Form I—Form II) of the structures modeled using PBESOL functional, with respect to pressure. (**B**): The change of unit cell edge “a” length obtained from calculations using PBE TS functional, with respect to pressure. Yellow circles—using Form I as initial; violet circles—using Form II as initial.

The next step of the analysis was the comparison of the energies obtained while applying the same pressure values and DFT functionals but using different initial structures (Forms I and II). Results are presented in Tables S4 and S5, Figure 4 and Figure 5. In those figures, the calculated unit cell lengths “a”, obtained starting either from Form I or II, have also been shown as they are relevant for the discussion below. The positive values of differences indicate that form II is energetically preferred.

Looking at Figure 4 and Figure 5, some similarities and differences between those results can be observed. For the lower pressure values (lower than 1.40 GPa and 3.20 GPa for PBE TS and PBESOL, respectively), the calculated differences between the energies were found to be very small. It is not surprising that for those calculations the transition of Form II into Form I has been observed. This is clearly visible, by looking at the right parts of those figures presenting the values of “a” lengths. The absolute value of the energy difference corresponds with the difference between the “a” values calculated using different Forms as the initial. Then, above a certain value of pressure (2.20 GPa and 3.50 GPa for PBE TS and PBESOL, respectively), the values of energy differences begin to increase monotonically, meaning, that according to both PBE TS and PBESOL calculations results, Form II is becoming more stable with the increase in pressure, which is in agreement with experimental observations. However, in the whole studied pressure range, the differences obtained using PBESOL were found to be negative, meaning that according to the PBESOL calculations Form I should be more energetically favorable than Form II under those conditions. Those results were not in agreement with experimental observations. However, for the results obtained using PBE TS, the change of the sign of the calculated differences was observed at a pressure higher than 5.50 GPa. Therefore, according to the calculations obtained using PBE TS functional, Form II should be more stable than Form I at higher pressure, which was confirmed experimentally. For the PBE TS calculations, if the difference between the forms was larger than 3 kJ/mol the transition of Form II into Form I was observed (at c.a. 2.00 GPa). In the case of PBESOL, the difference had to be larger than 6.60 kJ/mol to force the transition which occurred at 3.20 GPa.

The most important conclusion from the results observed and discussed above was that the results obtained using the PBE TS functional suggested that Form II should be more stable under increased pressure; however, the IPT was not observed during geometry optimization. This conclusion encouraged us to try to overcome the energy barrier between Form I and Form II using ab initio molecular dynamics (aiMD) which will be described in detail in Section 2.5.

**Figure 6:**
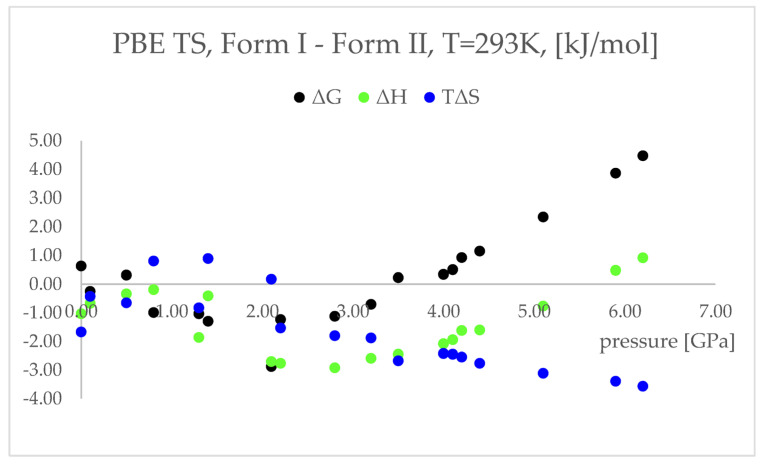
Differences between the thermodynamic parameters: free energy (ΔG, black dots) enthalpy (ΔH, green dots), temperature times entropy (TΔS, blue dots); Form I—Form II; of the structures modelled using PBE TS functional at 293 K, with respect to pressure.

More accurately, it is not the electronic energy difference discussed above, but the difference between the Gibbs free energy (ΔG) that decides which polymorphic form is more stable at certain conditions. Therefore, to obtain the thermodynamic parameters of the studied forms, the calculations of phonon density of states were performed. The results of those calculations are presented and discussed below (Figure 6, Table S6). The entropy (ΔS) values were multiplied by the temperature (T = 293 K) to facilitate the analysis, as ΔG = ΔH–TΔS.

The differences between the ΔH values were found to change similarly to the changes in energy values (Figure 4), suggesting that the IPT of Form I to Form II should be exothermic at pressures higher than 5.5 GPa. However, the differences between the TΔS in the pressure range 2.20–6.20 GPa were found to be negative and monotonically decreasing. This indicated that the studied pressure-induced IPT should be entropy-driven transformation. The calculated changes in the free energy (ΔG) suggested that the studied Forms should coexist at pressure range 3.5–4.1 GPa and above this pressure Form II should be more stable and dominant, which is in agreement with the experimental data.

Concluding this section, the geometry optimization and thermodynamic properties calculations enabled the prediction of Form II to Form I IPT that occurs upon decompression. Further, the free energy calculations results agreed with experimental observations, indicating that Form II is more stable at higher pressure. However, probably due to the large energy barrier between Form I and Form II at higher pressure, the geometry optimization of Form I did not result in obtaining Form II at any of the studied pressure values. In order to achieve this aim, the aiMD dynamics calculations were performed.

### 2.5. Ab Initio Molecular Dynamics Simulations

As stated above, the geometry optimization of Form I at increased pressure did not result in Form II, which was the major aim of this study. However, encouraged by the results of energy (Figure 4) and thermodynamic parameters (Figure 6) calculations, showing that Form II is indeed more stable at increased pressure, we decided to perform the computationally demanding ab initio molecular dynamics (aiMD) simulations. Due to the small differences between the energies of the studied Forms, even at 6.2 GPa, being in the order of 1 kJ/mol, we have not performed the “classical” molecular dynamics simulations based on the molecular mechanics’ framework as their accuracy was expected to be insufficient.

For the aiMD simulations, GGA PBE TS functional was chosen, opposite to PBESOL, and Form II was found to be energetically favorable (Figure 4) at increased pressure. The simulations were performed at T = 293 K and p = 6.20 GPa, as this was the largest value of pressure at which the structural information for CT was obtained and, at the same time, the difference between the energies of the studied Forms at this pressure was the largest, in favor of Form II (Figure 4).

**Figure 7:**
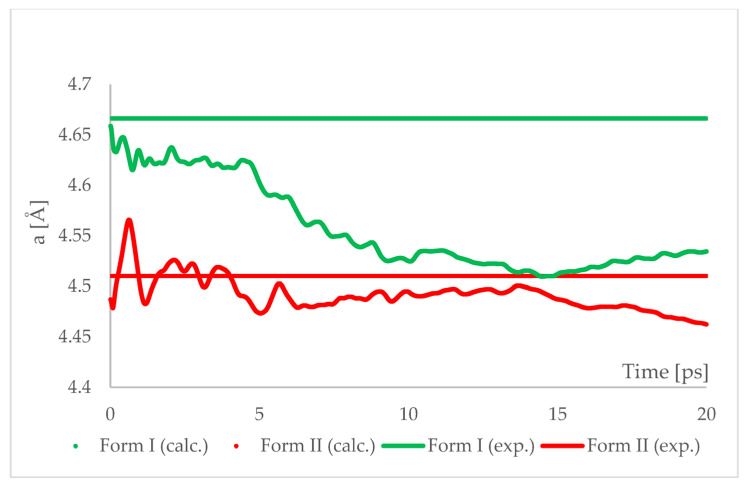
Running average of the unit cell edge length “a” obtained from aiMD simulation at T = 293 K and p = 6.2 GPa using PBE TS functional. Horizontal lines represent the experimental values.

**Figure 8:**
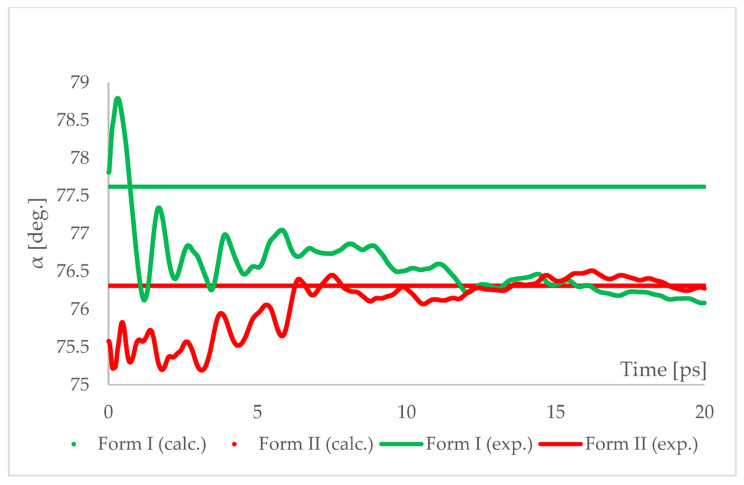
Running average of the unit cell angle “α” obtained from aiMD simulation at T = 293 K and p = 6.2 GPa using PBE TS functional. Horizontal lines represent the experimental values.

The geometry optimized at 6.20 GPa Forms I (QQQAUG09) and II (QQQAUG17) structures were used as starting for aiMD simulations. Form I was the obvious choice as the aim was to observe the IPT and obtain Form II. Additionally, simulations with the same parameters were performed also using Form II as initial for several reasons. First, we wanted to confirm that under those conditions no other phase transition would occur as well as to confirm the stability of this form under this pressure condition. Though experimentally Form II was found to be stable at 6.20 GPa, we wanted to ensure that the results of the calculations would be in agreement with this experimental observation. Secondly, since the introduction of kinetic energy associated with temperature always results in structural parameter fluctuations, it was necessary to determine the magnitude of such fluctuations. The results of aiMD are presented in Figure 7 and Figure 8 and Figures S1–S4.

The results of aiMD showed that it was a proper method to simulate the pressure-induced IPT of CT Form I into Form II. All the structural unit cell parameters of Form I changed into those of Form II during the simulation. Using previously discussed “a” edge as an example, the value of Form II exhibits only thermal fluctuations while the value of Form I decreases monotonically for the first 15 ps, reaching the experimental and computational values obtained for Form II. The simulation time needed for the structural parameters of Form I to convert into those of Form II was not common, i.e., in the case of angle α (Figure 8) it was shorter than for the edge “a”. Nevertheless, if only Form I is known, performing aiMD simulations at 6.20 GPa, preceded by the geometry optimization, would suggest that IPT may occur and would allow the estimation of the unit cell parameters of the new form. The reason why the aiMD simulations were required to observe the IPT that was not achieved in the geometry optimization is surely connected with the energy barrier between those two forms. The results of the thermodynamic calculations, presented in Figure 6, suggested that this transition is entropy-driven. Therefore, by adding kinetic energy in the aiMD simulations, it was possible for the studied system (Form I) to overcome this energy barrier and reach the deeper minimum (Form II). MD simulations are complementary to lattice-dynamical calculations in the sense that the latter are better suited to low temperatures, whereas the former are subject to ergodicity problems. Lattice dynamics are by definition limited to the (quasi)harmonic regime, while molecular dynamics naturally account for all the anharmonic effects occurring at high temperatures.

Therefore, answering the question stated in the title of this work, the DFT-based calculations can predict the pressure-induced IPT of CT, though only through the aiMD simulations.

## 3. Computational Methods

The density functional theory (DFT) calculations of geometry optimization, ab initio molecular dynamics (aiMD), phonon dispersion, and density of states were carried out with the CASTEP program [27] implemented in the Materials Studio 2017 software [28] using the plane wave pseudopotential formalism. On the fly generated (OTFG) norm-conserving pseudopotentials (NCP) were generated using the Koelling–Harmon (KH) scalar relativistic approach [29].

For comparison of the conformations of structures under investigation, the direct root mean square deviation (RMSD) of atomic positions for single molecules was calculated (Table 2) according to the Equation:

$$RMSD=\sqrt{\frac{\Sigma_{i}d_{i}^{2}}{n}}$$

where *d* is the distance between each of the n pairs of equivalent atoms in two optimally superposed structures.

To facilitate the analysis of the data in Table 2 a three-color scale was applied. In this scale, the 50th percentile (midpoint) was calculated, and the cell that holds this value was colored yellow. The cell that holds the minimum value was colored green, and the cell that holds the maximum value was colored red.

### 3.1. DFT Functionals and Dispersion Correction Methods

The Perdew–Burke–Ernzerhof (PBE) [30] pure or with Tkatchenko–Scheffler (TS) [20] or Gimme [31] dispersion correction, Perdew–Wang (PW91) [32] pure or with Ortmann–Bechstedt–Schmidt (OBS) [33] dispersion correction, revised Perdew–Burke–Ernzerhof (RPBE) [34], Wu–Cohen (WC) [35], solid-design version of the PBE (PBESOL) [36] exchange-correlation functionals, defined within the generalized gradient approximation (GGA) as well as the local exchange-correlation functional of Perdew and Zunger [37] with the parameterization of the numerical results of Ceperley and Alder [38] (LDA CA-PZ), with or without the OBS method of dispersion correction were used in the calculations.

### 3.2. Geometry Optimization

Geometry optimization was carried out using the Broyden−Fletcher−Goldfarb−Shanno (BFGS) [39] optimization scheme and smart method for finite basis set correction. The electronic parameters—kinetic energy cutoff for the plane waves (E_cut_) and number of Monkhorst–Pack k-points during sampling for a primitive cell Brillouin zone integration [40] were optimized and set to 990 eV and 3 × 3 × 2, respectively.

The experimental X-ray structure of chlorothiazide Form I (refcode QQQAUG09) and Form II (QQQAUG17) from the Cambridge Structure Database (CSD) were used as initial for calculations. During geometry optimization, all atom positions and the cell parameters were optimized, with no constraints. The convergence criteria were set at 5 × 10^−6^ eV/atom for the energy, 1 × 10^−2^ eV/Å for the interatomic forces, 2 × 10^−2^ GPa for the stresses, and 5 × 10^−4^ Å for the displacements. The fixed basis set quality method for the cell optimization calculations and the 5 × 10^−7^ eV/atom tolerance for SCF were used.

### 3.3. Thermodynamic Parameters Calculations

Phonon frequencies were obtained by diagonalization of dynamical matrices computed using linear response methodology (also known as density functional perturbation theory, DFPT) [41]. DFPT is the most commonly used ab initio calculation method of phonons. This method is different from a direct method since DFPT calculates the change in the Hamiltonian under a given perturbation of charge density or wavefunction, rather than directly displacing atoms in a direct method. The q-point separation parameter, which represents the average distance between Monkhorst–Pack mesh q-points used in the real space dynamical matrix calculations, was set to 0.05 Å^−1^. The convergence criterion for the force constants during a phonon properties run was set to 1 × 10^−5^ eV/Å^2^. This model was employed to calculate band structure, DOS, phonon spectrum, and phonon DOS properties. For the dispersion calculations, the separation between consecutive q-vectors on the reciprocal space path was set to 0.015 Å^−1^. For the DOS calculations, the 3 × 3 × 2 Monkhorst–Pack k-points grid has been chosen, resulting in the q-vector separation of 0.04 Å^−1^.

The results of a calculation of phonon spectra have been used to compute, in the quasi-harmonic approximation, zero-point vibrational energy (*E_zp_*), entropy (*S*), Gibbs free energy (*G*), and enthalpy (*H*) as functions of temperature, using the Formulas (1)–(4) below that are based on the work by Baroni et al. [41]. In those formula, ω represents phonon frequency, *F*(*ω*) represents the vibrational density of states for a phonon spectrum, *E_tot_* is the total electronic energy at 0 K, *k* is Boltzmann’s constant, and ħ is the Dirac’s constant.

(1) $$E_{zp}=\frac{1}{2}\int^{\;}F\left(\omega \right)\hbar \omega d\omega$$

(2) $$S\left(T\right)=k\left\{\int^{\;}\frac{\frac{\hbar \omega }{kT}}{\exp\left(\frac{\hbar \omega }{kT}\right)-1}F\left(\omega \right)d\omega -\int^{\;}F\left(\omega \right)\ln\left[1-\exp\left(-\frac{\hbar \omega }{kT}\right)\right]d\omega \right\}$$

(3) $$G\left(T\right)=E_{tot}+E_{zp}+kT\int^{\;}F\left(\omega \right)\ln\left[1-\exp\left(-\frac{\hbar \omega }{kT}\right)\right]d\omega +pV$$

(4) H=G+TS

### 3.4. Ab Initio Molecular Dynamic Simulations (aiMD)

Born–Oppenheimer ab initio molecular dynamics (aiMD) [42] simulations were run in CASTEP using an NPT ensemble maintained at a constant temperature of 293 K and pressure of 6.20 GPa, using Nosé thermostat, Parinello barostat, and PBE TS functional. The kinetic energy cutoff for the plane waves (*E_cut_*) was set to 990 eV and the integration time step was set to 0.5 fs. No symmetry constraints were applied during the simulations. The total time of the simulation was set to 20 ps.

## 4. Conclusions

In this work, the pressure-induced IPT of chlorothiazide was studied using DFT methods. First, the accuracy of the calculations using different DFT functionals was evaluated, resulting in the choice of the PBE TS and PBESOL for future calculations. Then, the geometry optimization calculations of Form I and Form II at all experimentally studied pressure conditions were performed. It was observed that the choice of DFT functional had a significant influence on the received results. In particular, the dispersion correction (TS) was found to be crucial for achieving accurate results, and thus for the thermodynamic and aiMD calculations only the PBE TS functional has been chosen. Through the geometry optimization, the IPT of Form II into Form I upon decompression was achieved, however, the opposite pressure-induced transition was not observed, regardless of the chosen functional. However, both the comparison of the electronic energies and chosen thermodynamic parameters (ΔG, ΔH, ΔS) indicated that Form II is more stable at the increased pressure. Finally, in order to observe the pressure-induced IPT of Form I into Form II, ab initio molecular dynamics simulations were successfully applied.

Since the DFT calculations enabled to predict the IPT of CT, we will continue to study the other IPT using the methodology described in this work. We hope that such an approach, when successfully validated on the reasonable number of examples, will be used in the future as a screening method to predict the isosymmetric phase transition, lowering the costs and increasing the efficiency of the studies designed for searching of new polymorphic forms.

## Figures and Tables

**Table 1:** Structural parameters of crystal forms of chlorothiazide. Data for Form II were bolded to increase the clarity.

Form	p [GPa]	a [Å]	b [Å]	c [Å]	α [^o^]	β [^o^]	γ [^o^]	V [Å ^3^]	XRD
I	0.0	4.886	6.405	8.985	74.05	83.56	80.54	265.01	PXRD
I	0.0	4.875	6.401	8.980	74.05	83.54	80.47	264.01	SCXRD
I	0.1	4.873	6.413	8.941	74.07	83.71	80.59	263.50	PXRD
I	0.5	4.835	6.310	8.895	74.62	84.12	80.67	256.84	SCXRD
I	0.8	4.812	6.278	8.829	75.09	84.07	80.94	253.18	PXRD
I	1.3	4.760	6.146	8.737	76.06	84.56	81.25	244.06	SCXRD
I	1.4	4.768	6.185	8.767	75.66	84.53	81.24	246.43	PXRD
I	2.1	4.728	6.050	8.684	76.54	84.73	81.67	238.02	SCXRD
I	2.2	4.734	6.074	8.691	76.30	84.83	81.70	239.29	PXRD
I	2.8	4.718	5.998	8.656	76.64	84.89	82.05	235.11	PXRD
I	3.2	4.678	5.910	8.593	77.22	85.05	82.54	228.90	PXRD
I	3.5	4.693	5.901	8.599	77.48	84.97	82.35	229.53	SCXRD
I	4.0	4.676	5.812	8.543	77.95	85.11	82.77	224.41	SCXRD
I	4.1	4.681	5.895	8.587	77.34	85.12	82.36	228.31	PXRD
I	4.2	4.680	5.848	8.592	77.63	84.70	81.77	226.34	PXRD
**II**	**4.2**	**4.529**	**5.957**	**8.540**	**76.52**	**76.52**	**83.27**	**216.35**	**PXRD**
**II**	**4.4**	**4.510**	**5.929**	**8.503**	**76.53**	**85.62**	**83.20**	**218.91**	**SCXRD**
**II**	**5.1**	**4.483**	**5.893**	**8.465**	**76.23**	**85.84**	**83.28**	**215.16**	**SCXRD**
I	5.1	4.696	5.804	8.538	77.98	84.85	81.80	224.37	PXRD
**II**	**5.1**	**4.524**	**5.933**	**8.524**	**76.64**	**85.54**	**83.48**	**220.50**	**PXRD**
**II**	**5.9**	**4.461**	**5.859**	**8.427**	**75.99**	**86.06**	**83.35**	**211.77**	**SCXRD**
I	6.2	4.666	5.793	8.618	77.62	85.25	82.03	224.58	PXRD
**II**	**6.2**	**4.510**	**5.897**	**8.472**	**76.31**	**85.65**	**83.30**	**216.80**	**PXRD**

Form—either I or II polymorph, according to [21]; p—pressure at which the structure was studied; a, b, c, α, β, γ, V—unit cell dimensions; XRD-type of X-ray diffraction experiment applied to obtain the structure information (PXRD—powder X-ray diffraction; SCXRD—single-crystal X-ray diffraction).

**Table 2:** **A.** RMSD of the structures at 0 GPa. **B.** RMSD of the structures at 0.5 GPa. **C.** RMSD of the structures at 1.3 GPa. **D.** RMSD of the structures at 2.1 GPa. **E.** RMSD of the structures at 3.5 GPa. **F.** RMSD of the structures at 4.0 GPa. **G.** RMSD of the structures at 4.4 GPa. **H.** RMSD of the structures at 5.1 GPa. **I.** RMSD of the structures at 5.9 GPa. To facilitate the analysis of the data in Table 2 a three-color scale was applied. In this scale, the 50th percentile (midpoint) was calculated, and the cell that holds this value was colored yellow. The cell that holds the minimum value was colored green, and the cell that holds the maximum value was colored red.

A p = 0 GPa, Form I	Exp	PBESOLII	PBESOL I	PBE TS II	PBE TS I
Exp	0	0.1724	0.1249	0.1159	0.1010
PBESOL II	0.1724	0	0.0854	0.1138	0.1467
PBESOL I	0.1249	0.0854	0	0.0836	0.0862
PBE TS II	0.1159	0.1138	0.0836	0	0.0572
PBE TS I	0.1010	0.1467	0.0862	0.0572	0
**B** **p = 0.5 GPa, Form I**	**Exp**	**PBESOLII**	**PBESOL I**	**PBE TS II**	**PBE TS I**
Exp	0	0.1398	0.1079	0.1028	0.1039
PBESOL II	0.1398	0	0.0663	0.1153	0.1257
PBESOL I	0.1079	0.0663	0	0.0585	0.0653
PBE TS II	0.1028	0.1153	0.0585	0	0.0209
PBE TS I	0.1039	0.1257	0.0653	0.0209	0
**C** **p = 1.3 GPa, Form I**	**Exp**	**PBESOL II**	**PBESOL I**	**PBE TS II**	**PBE TS I**
Exp	0	0.1115	0.1093	0.1447	0.1070
PBESOL II	0.1115	0	0.0241	0.1255	0.0478
PBESOL I	0.1093	0.0241	0	0.1024	0.0367
PBE TS II	0.1447	0.1255	0.1024	0	0.0921
PBE TS I	0.1070	0.0478	0.0367	0.0921	0
**D** **p = 2.1 GPa, Form I**	**Exp**	**PBESOL II**	**PBESOL I**	**PBE TS II**	**PBE TS I**
exp	0	0.1192	0.1166	0.1703	0.1169
PBESOL II	0.1192	0	0.0169	0.1635	0.0325
PBESOL I	0.1166	0.0169	0	0.1478	0.0230
PBE TS II	0.1703	0.1635	0.1478	0	0.1428
PBE TS I	0.1169	0.0325	0.0230	0.1428	0
**E** **p = 3.5 GPa, Form I**	**Exp**	**PBESOL II**	**PBESOL I**	**PBE TS II**	**PBE TS I**
exp	0	0.2278	0.1224	0.2566	0.1212
PBESOL II	0.2278	0	0.2165	0.0407	0.2120
PBESOL I	0.1224	0.2165	0	0.2507	0.0149
PBE TS II	0.2566	0.0407	0.2507	0	0.2456
PBE TS I	0.1212	0.2120	0.0149	0.2456	0
**F** **p = 4.0 GPa, Form I**	**Exp**	**PBESOL II**	**PBESOL I**	**PBE TS II**	**PBE TS I**
exp	0	0.2646	0.1231	0.2767	0.1254
PBESOL II	0.2646	0	0.2577	0.0262	0.2738
PBESOL I	0.1231	0.2577	0	0.2738	0.0215
PBE TS II	0.2767	0.0262	0.2738	0	0.2898
PBE TS I	0.1254	0.2738	0.0215	0.2898	0
**G** **p = 4.4 GPa, Form II**	**Exp**	**PBESOL II**	**PBESOL I**	**PBE TS II**	**PBE TS I**
exp	0	0.1408	0.2952	0.1422	0.3058
PBESOL II	0.1408	0	0.2846	0.0226	0.2973
PBESOL I	0.2952	0.2846	0	0.3002	0.0194
PBE TS II	0.1422	0.0226	0.3002	0	0.3127
PBE TS I	0.3058	0.2973	0.0194	0.3127	0
**H** **p = 5.1 GPa, Form II**	**Exp**	**PBESOL II**	**PBESOL I**	**PBE TS II**	**PBE TS I**
exp	0	0.1411	0.3171	0.1402	0.3243
PBESOL II	0.1411	0	0.3125	0.0216	0.3216
PBESOL I	0.3171	0.3125	0	0.3245	0.0179
PBE TS II	0.1402	0.0216	0.3245	0	0.3334
PBE TS I	0.3243	0.3216	0.0179	0.3334	0
**I** **p = 5.9 GPa, Form II**	**Exp**	**PBESOL II**	**PBESOL I**	**PBE TS II**	**PBE TS I**
exp	0	0.1453	0.3372	0.1425	0.3419
PBESOL II	0.1453	0	0.3361	0.0194	0.3421
PBESOL I	0.3372	0.3361	0	0.3462	0.0182
PBE TS II	0.1425	0.0194	0.3462	0	0.3520
PBE TS I	0.3419	0.3421	0.0182	0.3520	0

## Data Availability

The data presented in this study are available on request from the corresponding author.

## Supplementary Materials

The following are available online at https://www.mdpi.com/article/10.3390/ijms221810100/s1.
