# Supplementary material for: Can We Predict the Isosymmetric Phase Transition? Application of DFT Calculations to Study the Pressure Induced Transformation of Chlorothiazide
Source: Int J Mol Sci. 2021 Sep 18;22(18):10100. doi: 10.3390/ijms221810100 (PMC8465122; doi:10.3390/ijms221810100)
Supplement: Supplementary file 1 [file ijms-22-10100-s001.zip › ijms-1337062-supplementary.pdf]

International Journal of *Molecular Sciences*

MDPI

Article

Can we predict the isosymmetric phase transition? Application of DFT calculations to study the pressure induced transformation of chlorothiazide.

Łukasz Szeleszczuk*[1], Anna Helena Mazurek[2], Katarzyna Milcarz[1], Ewa Napiórkowska[1], Dariusz Maciej Pisklak[1]

1 Department of Physical Chemistry, Chair and Department of Physical Pharmacy and Bioanalysis, Faculty of Pharmacy, Medical University of Warsaw, Banacha 1 str., 02-093 Warsaw, Poland; lszeleszczuk@wum.edu.pl (Ł.S.); kmilcarz@wum.edu.pl (K.M.); enapiorkowska@wum.edu.pl (E.N.); dpisklak@wum.edu.pl (D.M.P.),

2 Department of Physical Chemistry, Chair and Department of Physical Pharmacy and Bioanalysis, Faculty of Pharmacy, Doctoral School, Medical University of Warsaw, Banacha 1 str., 02-093 Warsaw, Poland; amazurek1@wum.edu.pl (A.H.M.)

* Correspondence: lszeleszczuk@wum.edu.pl; Tel.: +48-501-255-121

Supplementary materials

Table S1. Experimental (refcode: QQQAUG09) and calculated unit cell dimensions of Form I of chlorothiazide. The differences (Δ) between the corresponding experimental and calculated values were provided to facilitate the assessment of the accuracy of calculations.

DFT functional	a [Å]	Δa [Å]	b [Å]	Δb [Å]	c [Å]	Δc [Å]	α [°]	Δα [°]	β [°]	Δβ [°]	γ [°]	Δγ [°]	V[Å^3]	ΔV[Å^3]
Experimental	4.875		6.401		8.980		74.05		83.54		80.47		264.01	
GGA PBESOL	4.980	-0.105	6.691	-0.290	9.316	-0.336	70.38	3.67	79.94	3.60	78.41	2.06	282.04	-18.04
LDA-CA-PZ	4.738	0.136	6.311	0.090	8.737	0.243	73.84	0.21	83.57	-0.03	79.35	1.12	245.07	18.94
LDA-CA-PZ-OBS	4.658	0.217	5.978	0.423	8.459	0.521	76.01	-1.96	84.98	-1.44	80.70	-0.23	224.68	39.33
GGA PBE	5.205	-0.330	6.804	-0.403	9.697	-0.717	69.98	4.07	78.24	5.30	78.72	1.74	309.80	-45.79
GGA PBE TS	4.917	-0.042	6.405	-0.004	8.964	0.016	74.14	-0.09	83.79	-0.25	80.84	-0.38	266.53	-2.53
GGA PBE Grimme	4.938	-0.063	6.484	-0.083	8.914	0.066	73.21	0.84	83.92	-0.38	79.72	0.74	267.35	-3.34
GGA RPBE	5.610	-0.736	6.972	-0.570	10.285	-1.305	69.28	4.77	76.37	7.17	79.22	1.25	359.20	-95.20
GGA PW91	5.291	-0.416	6.725	-0.324	9.992	-1.012	70.15	3.90	75.61	7.93	80.33	0.13	319.30	-55.30
GGA PW91 OBS	4.942	-0.067	6.503	-0.101	8.937	0.043	72.75	1.30	83.66	-0.12	79.15	1.31	267.73	-3.72
GGA WC	5.251	-0.377	6.598	-0.196	10.113	-1.133	69.59	4.46	72.54	11.00	79.88	0.58	308.37	-44.37

Table S2. Calculated, at various pressure, unit cell dimensions of chlorothiazide using Form I (QQQAUG09) or Form II (QQQAUG17) structures as initial and GGA PBESOL functional.

DFT functional	Pressure [GPa]	Initial structure	a [Å]	b [Å]	c [Å]	α [°]	β [°]	γ [°]	V[$Å^3$]
GGA PBESOL	0.00	QQQAUG09	4.980	6.691	9.316	70.38	79.94	78.41	282.04
GGA PBESOL	0.10	QQQAUG09	4.955	6.651	9.251	70.94	80.49	78.57	278.55
GGA PBESOL	0.50	QQQAUG09	4.885	6.505	8.992	72.76	82.33	78.91	265.43
GGA PBESOL	0.80	QQQAUG09	4.852	6.443	8.903	73.23	83.07	79.28	259.94
GGA PBESOL	1.30	QQQAUG09	4.807	6.333	8.775	73.96	83.75	79.87	251.20
GGA PBESOL	1.40	QQQAUG09	4.798	6.314	8.761	74.15	83.82	79.96	249.95
GGA PBESOL	2.10	QQQAUG09	4.763	6.171	8.665	75.00	84.25	80.73	241.61
GGA PBESOL	2.20	QQQAUG09	4.760	6.145	8.656	75.11	84.28	80.94	240.42
GGA PBESOL	2.80	QQQAUG09	4.731	6.060	8.603	75.64	84.55	81.28	235.09
GGA PBESOL	3.20	QQQAUG09	4.708	6.032	8.557	75.86	84.69	81.46	232.07
GGA PBESOL	3.50	QQQAUG09	4.702	5.978	8.543	76.22	84.76	81.59	229.76
GGA PBESOL	4.00	QQQAUG09	4.683	5.922	8.512	76.52	84.90	81.89	226.37
GGA PBESOL	4.10	QQQAUG09	4.681	5.915	8.502	76.46	84.89	82.05	225.75
GGA PBESOL	4.20	QQQAUG09	4.676	5.903	8.500	76.65	84.91	81.94	225.16
GGA PBESOL	4.40	QQQAUG09	4.675	5.881	8.482	76.74	84.97	82.08	223.96
GGA PBESOL	5.10	QQQAUG09	4.652	5.818	8.444	77.12	85.06	82.39	220.00
GGA PBESOL	5.90	QQQAUG09	4.635	5.739	8.411	77.56	85.16	82.79	216.00
GGA PBESOL	6.20	QQQAUG09	4.625	5.724	8.394	77.68	85.22	82.82	214.62
GGA PBESOL	0.00	QQQAUG17	5.050	6.636	9.596	70.78	77.45	81.02	292.76
GGA PBESOL	0.10	QQQAUG17	4.951	6.647	9.416	71.33	79.20	80.76	284.65
GGA PBESOL	0.50	QQQAUG17	4.858	6.609	9.092	72.08	81.98	79.78	270.68
GGA PBESOL	0.80	QQQAUG17	4.842	6.458	8.927	73.22	82.85	79.17	260.49
GGA PBESOL	1.30	QQQAUG17	4.811	6.321	8.792	73.74	83.63	80.16	251.33
GGA PBESOL	1.40	QQQAUG17	4.808	6.288	8.773	73.96	83.70	80.31	249.78
GGA PBESOL	2.10	QQQAUG17	4.770	6.158	8.663	74.97	84.24	80.81	241.36
GGA PBESOL	2.20	QQQAUG17	4.755	6.162	8.652	75.04	84.35	80.78	240.62
GGA PBESOL	2.80	QQQAUG17	4.732	6.059	8.604	75.62	84.51	81.35	235.15
GGA PBESOL	3.20	QQQAUG17	4.710	6.030	8.550	75.97	84.76	81.34	231.91
GGA PBESOL	3.50	QQQAUG17	4.568	6.008	8.624	78.02	84.41	83.12	228.78
GGA PBESOL	4.00	QQQAUG17	4.537	5.951	8.602	77.43	84.63	83.39	224.20
GGA PBESOL	4.10	QQQAUG17	4.536	5.958	8.585	77.76	84.69	83.25	224.18
GGA PBESOL	4.20	QQQAUG17	4.525	5.939	8.586	77.13	84.80	83.37	222.52
GGA PBESOL	4.40	QQQAUG17	4.516	5.926	8.570	77.05	84.89	83.38	221.16
GGA PBESOL	5.10	QQQAUG17	4.488	5.893	8.515	76.52	85.36	83.34	216.79
GGA PBESOL	5.90	QQQAUG17	4.461	5.859	8.452	76.34	85.70	83.24	212.56
GGA PBESOL	6.20	QQQAUG17	4.448	5.844	8.439	76.26	85.81	83.29	211.06

Table S3. Calculated, at various pressure, unit cell dimensions of chlorothiazide using Form I (QQQAUG09) or Form II (QQQAUG17) structures as initial and GGA PBE TS functional.

DFT functional	Pressure [GPa]	Initial structure	a [Å]	b [Å]	c [Å]	α [°]	β [°]	γ [°]	V[Å³]
GGA PBE TS	0.00	QQQAUG09	4.917	6.405	8.964	74.14	83.79	80.84	266.53
GGA PBE TS	0.10	QQQAUG09	4.906	6.450	8.931	74.09	83.97	81.07	266.97
GGA PBE TS	0.50	QQQAUG09	4.866	6.365	8.860	74.69	84.22	81.24	260.23
GGA PBE TS	0.80	QQQAUG09	4.841	6.325	8.824	74.83	84.33	81.28	256.47
GGA PBE TS	1.30	QQQAUG09	4.805	6.248	8.762	75.56	84.57	81.46	250.80
GGA PBE TS	1.40	QQQAUG09	4.802	6.230	8.758	75.43	84.54	81.52	249.70
GGA PBE TS	2.10	QQQAUG09	4.772	6.115	8.708	75.99	84.67	81.91	243.04
GGA PBE TS	2.20	QQQAUG09	4.772	6.093	8.707	76.03	84.62	82.09	242.25
GGA PBE TS	2.80	QQQAUG09	4.752	6.015	8.667	76.29	84.66	82.36	237.50
GGA PBE TS	3.20	QQQAUG09	4.731	5.991	8.630	76.60	84.84	82.26	234.84
GGA PBE TS	3.50	QQQAUG09	4.723	5.949	8.621	76.78	84.82	82.45	232.78
GGA PBE TS	4.00	QQQAUG09	4.719	5.882	8.595	76.86	84.77	82.91	229.61
GGA PBE TS	4.10	QQQAUG09	4.717	5.873	8.587	76.89	84.76	83.00	228.98
GGA PBE TS	4.20	QQQAUG09	4.704	5.900	8.560	77.06	85.04	82.66	228.81
GGA PBE TS	4.40	QQQAUG09	4.710	5.843	8.572	77.03	84.78	83.14	227.31
GGA PBE TS	5.10	QQQAUG09	4.695	5.777	8.539	77.40	84.82	83.42	223.62
GGA PBE TS	5.90	QQQAUG09	4.677	5.714	8.504	77.74	84.87	83.64	219.85
GGA PBE TS	6.20	QQQAUG09	4.671	5.693	8.490	77.91	84.89	83.66	218.55
GGA PBE TS	0.00	QQQAUG17	4.886	6.489	8.967	75.05	83.92	81.52	270.13
GGA PBE TS	0.10	QQQAUG17	4.884	6.478	8.930	74.68	84.09	81.11	267.77
GGA PBE TS	0.50	QQQAUG17	4.863	6.372	8.853	75.01	84.28	81.14	260.51
GGA PBE TS	0.80	QQQAUG17	4.841	6.320	8.821	75.09	84.36	81.25	256.53
GGA PBE TS	1.30	QQQAUG17	4.761	6.256	8.781	76.84	84.51	81.73	250.87
GGA PBE TS	1.40	QQQAUG17	4.799	6.233	8.749	75.76	84.61	81.32	249.67
GGA PBE TS	2.10	QQQAUG17	4.698	6.177	8.713	77.27	84.73	82.01	243.20
GGA PBE TS	2.20	QQQAUG17	4.679	6.161	8.724	77.31	84.59	82.35	242.11
GGA PBE TS	2.80	QQQAUG17	4.635	6.098	8.696	76.85	84.72	82.64	236.31
GGA PBE TS	3.20	QQQAUG17	4.609	6.071	8.671	76.89	84.82	82.58	233.36
GGA PBE TS	3.50	QQQAUG17	4.592	6.057	8.647	76.53	84.97	82.58	231.02
GGA PBE TS	4.00	QQQAUG17	4.566	6.032	8.610	76.29	85.19	82.53	227.62
GGA PBE TS	4.10	QQQAUG17	4.561	6.027	8.607	76.22	85.21	82.51	227.01
GGA PBE TS	4.20	QQQAUG17	4.554	6.017	8.609	76.10	85.26	82.64	226.33
GGA PBE TS	4.40	QQQAUG17	4.546	6.009	8.592	75.89	85.38	82.60	224.99
GGA PBE TS	5.10	QQQAUG17	4.520	5.982	8.539	75.59	85.65	82.42	221.03
GGA PBE TS	5.90	QQQAUG17	4.492	5.946	8.499	75.47	85.88	82.49	217.28
GGA PBE TS	6.20	QQQAUG17	4.481	5.938	8.478	75.43	86.00	82.41	215.90

Table S4. Calculated, at various pressure, energies [kJ/mol] of chlorothiazide using Form I (QQQAUG09) or Form II (QQQAUG17) structures as initial and GGA PBE TS functional.

DFT functional	Pressure [GPa]	Energy [kJ/mol]		
		QQQUG09 Form I	QQQAUG17 Form II	Δ (Form I – Form II)
GGA PBE TS	0.00	-465665.06	-465663.77	-1.29
GGA PBE TS	0.10	-465649.28	-465648.34	-0.94
GGA PBE TS	0.50	-465586.49	-465586.10	-0.38
GGA PBE TS	0.80	-465540.29	-465540.14	-0.15
GGA PBE TS	1.30	-465464.53	-465462.47	-2.06
GGA PBE TS	1.40	-465449.74	-465449.48	-0.25
GGA PBE TS	2.10	-465346.93	-465344.26	-2.67
GGA PBE TS	2.20	-465332.47	-465329.49	-2.98
GGA PBE TS	2.80	-465246.57	-465243.61	-2.96
GGA PBE TS	3.20	-465190.06	-465187.48	-2.58
GGA PBE TS	3.50	-465148.17	-465145.83	-2.34
GGA PBE TS	4.00	-465079.22	-465077.28	-1.94
GGA PBE TS	4.10	-465065.52	-465063.68	-1.85
GGA PBE TS	4.20	-465051.63	-465050.09	-1.54
GGA PBE TS	4.40	-465024.60	-465023.09	-1.51
GGA PBE TS	5.10	-464930.22	-464929.68	-0.54
GGA PBE TS	5.90	-464824.14	-464824.81	0.67
GGA PBE TS	6.20	-464784.80	-464785.94	1.14

Table S5. Calculated, at various pressure, energies [kJ/mol] of chlorothiazide using Form I (QQQAUG09) or Form II (QQQAUG17) structures as initial and GGA PBESOL functional.

DFT functional	Pressure [GPa]	Energy [kJ/mol]		
		QQQUG09 Form I	QQQAUG17 Form II	Δ (Form I – Form II)
GGA PBESOL	0.00	-464413.36	-464413.85	0.49
GGA PBESOL	0.10	-464396.64	-464396.48	-0.16
GGA PBESOL	0.50	-464331.70	-464330.57	-1.13
GGA PBESOL	0.80	-464284.58	-464284.40	-0.18
GGA PBESOL	1.30	-464208.42	-464208.51	0.09
GGA PBESOL	1.40	-464193.41	-464193.55	0.15
GGA PBESOL	2.10	-464090.72	-464090.81	0.09
GGA PBESOL	2.20	-464076.32	-464076.30	-0.02
GGA PBESOL	2.80	-463990.95	-463990.98	0.04
GGA PBESOL	3.20	-463935.04	-463935.04	0.00
GGA PBESOL	3.50	-463893.55	-463886.94	-6.60
GGA PBESOL	4.00	-463825.32	-463818.81	-6.51
GGA PBESOL	4.10	-463811.80	-463805.46	-6.35
GGA PBESOL	4.20	-463798.28	-463791.95	-6.33
GGA PBESOL	4.40	-463771.40	-463765.32	-6.08
GGA PBESOL	5.10	-463678.40	-463673.40	-5.00
GGA PBESOL	5.90	-463573.97	-463570.57	-3.40
GGA PBESOL	6.20	-463535.31	-463532.56	-2.75

Table S6. Calculated, using GGA PBE TS functional, at various pressure and T=293 K, differences between the chosen thermodynamic properties of Form I (QQQAUG09) and Form II (QQQAUG17). ΔH – enthalpy; ΔG - Gibbs free energy; TΔS - entropy multiplied by temperature (293K).

Pressure [GPa]	QQQAUG09 (Form I) – QQQAUG (Form II) [kJ/mol]		
	ΔH	ΔG	TΔS
0.00	-1.04	0.63	-1.67
0.10	-0.67	-0.25	-0.42
0.50	-0.34	0.31	-0.65
0.80	-0.19	-0.99	0.80
1.30	-1.86	-1.04	-0.83
1.40	-0.40	-1.29	0.89
2.10	-2.70	-2.88	0.17
2.20	-2.76	-1.24	-1.53
2.80	-2.92	-1.12	-1.80
3.20	-2.58	-0.70	-1.88
3.50	-2.44	0.23	-2.67
4.00	-2.08	0.34	-2.42
4.10	-1.94	0.50	-2.45
4.20	-1.62	0.93	-2.54
4.40	-1.60	1.15	-2.75
5.10	-0.76	2.35	-3.11
5.90	0.48	3.87	-3.39
6.20	0.92	4.48	-3.56

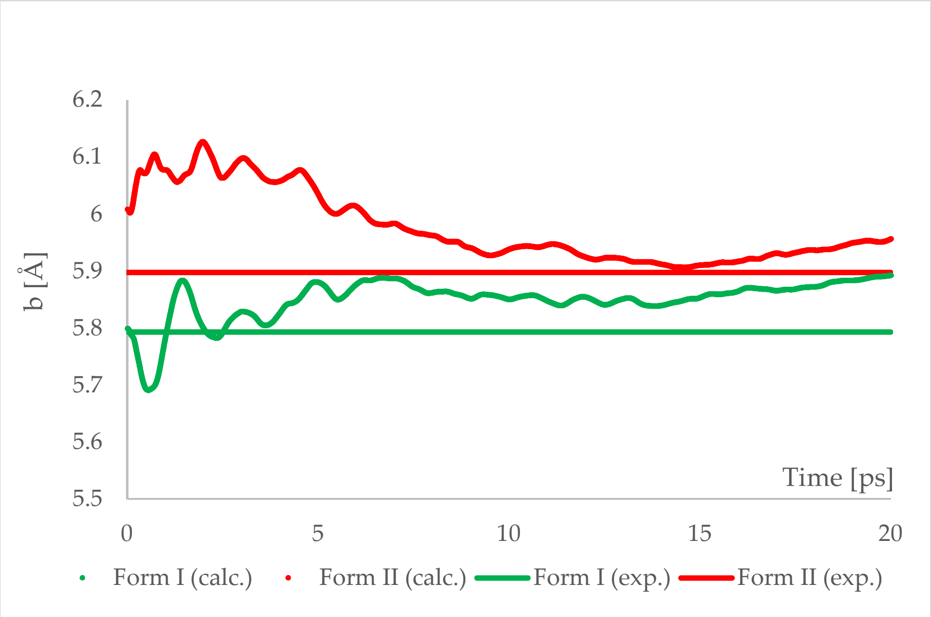

Figure S1. Running average of the unit cell length "b" obtained from aiMD simulation at T=293 K and p=6.2 GPa using PBE TS functional. Horizontal lines represent the experimental values.

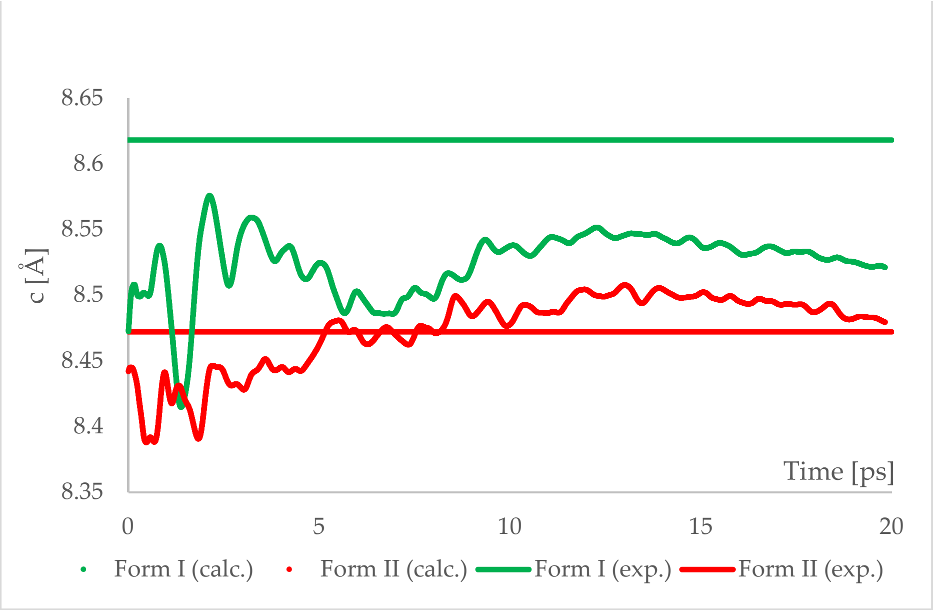

Figure S2. Running average of the unit cell length "c" obtained from aiMD simulation at T=293 K and p=6.2 GPa using PBE TS functional. Horizontal lines represent the experimental values.

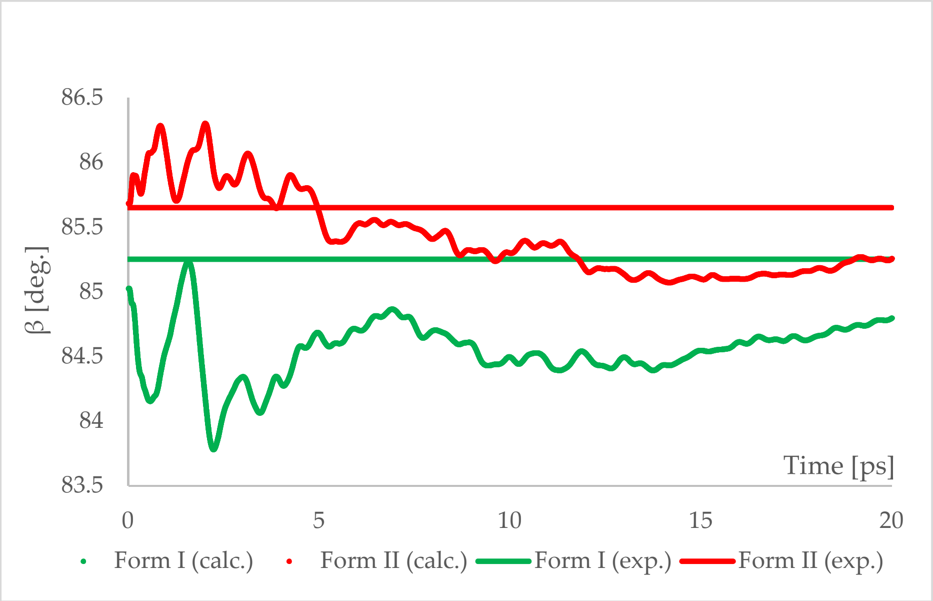

Figure S3. Running average of the unit cell angle "β" obtained from aiMD simulation at T=293 K and p=6.2 GPa using PBE TS functional. Horizontal lines represent the experimental values.

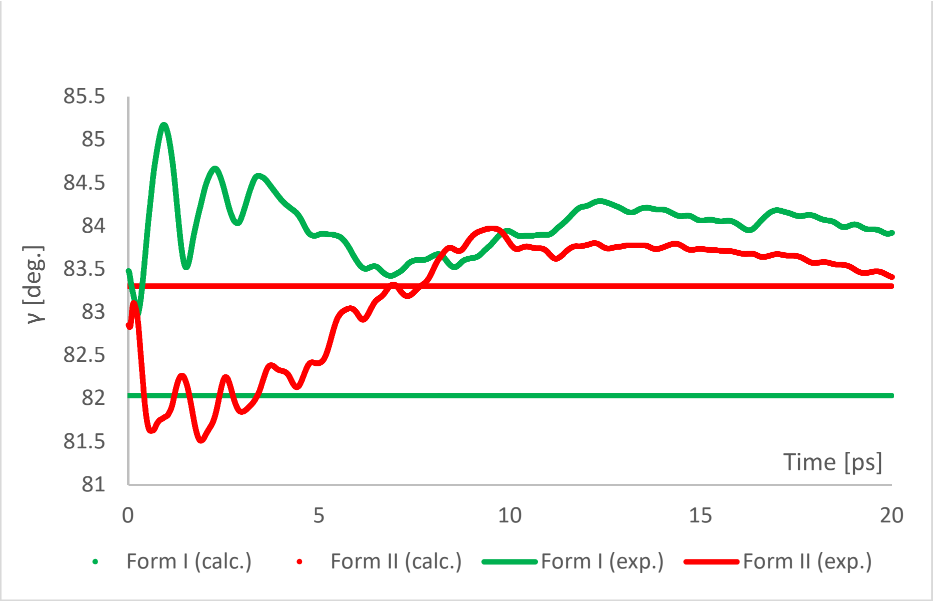

Figure S4. Running average of the unit cell angle "γ" obtained from aiMD simulation at T=293 K and p=6.2 GPa using PBE TS functional. Horizontal lines represent the experimental values.
